# Long-term oncological and surgical outcomes after Video Endoscopic Inguinal Lymphadenectomy (VEIL) in patients with penile cancer

**DOI:** 10.1590/S1677-5538.IBJU.2023.0065

**Published:** 2023-06-20

**Authors:** Marcos Tobias-Machado, Antonio A. Ornellas, Alexandre K. Hidaka, Luis G. Medina, Pablo A. L. Mattos, Ruben S. Besio, Diego Abreu, Pedro R. Castro, Ricardo H. Nishimoto, Juan Astigueta, Aurus Dourado, Roberto D. Machado, Wesley J. Magnabosco, Victor Corona-Montes, Gustavo M. Villoldo, Hamilton C. Zampolli, Anis Taha, Pericles R. Auad, Eliney F. Faria, Paulo B. O. Arantes, Alessandro Tavares, Francisco S. M. S. Nascimento, Eder S. Brazão, Maurício M. Rocha, Walter H. Costa, Vinicius Panico, Leonardo O. Reis, Roberto J. Almeida-Carrera, Rafael C. Silva, Stênio C. Zequi, José R. R. Calixto, Rene Sotelo

**Affiliations:** 1 Instituto do Cancer Arnaldo Vieira de Carvalho São Paulo SP Brasil Instituto do Cancer Arnaldo Vieira de Carvalho, São Paulo, SP, Brasil; 2 Centro Universitário Faculdade de Medicina do ABC Santo André SP Brasil Centro Universitário Faculdade de Medicina do ABC - FMABC, Santo André, SP, Brasil; 3 Instituto Nacional do Câncer Rio de Janeiro RJ Brasil Instituto Nacional do Câncer - INCA, Rio de Janeiro, RJ, Brasil; 4 University of Southern California Keck School of Medicine USC Institute of Urology Los Angeles CA USA USC Institute of Urology, Keck School of Medicine, University of Southern California, Los Angeles, CA, USA; 5 Hospital São Marcos Teresina PI Brasil Associação Piauiense de Combate ao Câncer - Hospital São Marcos, Teresina, PI, Brasil; 6 Hospital Pasteur Montevideo Uruguay Hospital Pasteur, Montevideo, Uruguay; 7 Hospital Felício Rocho Belo Horizonte MG Brasil Hospital Felício Rocho, Belo Horizonte, MG, Brasil; 8 Hospital Madre Teresa Belo Horizonte MG Brasil Hospital Madre Teresa, Belo Horizonte, MG, Brasil; 9 Universidad Privada Antenor Orrego Instituto Regional de Enfermedades Neoplásicas Norte Trujillo Perú Universidad Privada Antenor Orrego, Instituto Regional de Enfermedades Neoplásicas Norte, Trujillo, Perú; 10 Hospital de Amor Barretos SP Brasil Hospital de Amor, Barretos, Barretos, SP, Brasil; 11 Hospital Câncer de Barretos Barretos SP Brasil Hospital Câncer de Barretos, Barretos, SP, Brasil; 12 Hospital General de México “Dr. Eduardo Liceaga” Mexico city México Hospital General de México “Dr. Eduardo Liceaga”, Mexico city, México; 13 Alexander Fleming Institute Buenos Aires Argentina Alexander Fleming Institute, Buenos Aires, Argentina; 14 Universidade de São Paulo Hospital das Clínicas São Paulo SP Brasil Hospital das Clínicas, Universidade de São Paulo, São Paulo, SP, Brasil; 15 Universidade Federal do Maranhão Maranhão MA Brasil Universidade Federal do Maranhão - UFMA, Maranhão, MA, Brasil; 16 AC Camargo Cancer Center São Paulo SP Brasil AC Camargo Cancer Center, São Paulo, SP, Brasil; 17 National Institute for Science and Technology in Ocogenomic and Therapeutic Innovation AC Camargo Cancer Center São Paulo SP Brasil National Institute for Science and Technology in Ocogenomic and Therapeutic Innovation INCIT/INOTE AC Camargo Cancer Center, São Paulo, SP, Brasil; 18 Hospital Cancer de Londrina Londrina PR Brasil Hospital Cancer de Londrina, Londrina, PR, Brasil; 19 Universidade de Campinas Escola de Ciências Médicas Campinas SP Brasil UroScience, Escola de Ciências Médicas, Universidade de Campinas – UNICAMP, Campinas, SP, Brasil; 20 Pontifícia Universidade Católica de Campinas Campinas SP Brasil Pontifícia Universidade Católica de Campinas, Campinas - PUC, SP, Brasil; 21 Hospital Carlos Andrade Marin Quito Equador Hospital Carlos Andrade Marin, Quito, Equador

**Keywords:** Minimally Invasive Surgical Procedures, Laparoscopy, Penis

## Abstract

**Objective:**

To report outcomes from the largest multicenter series of penile cancer patients undergoing video endoscopic inguinal lymphadenectomy (VEIL).

**Materials and Methods:**

Retrospective multicenter analysis. Authors of 21 centers from the Penile Cancer Collaborative Coalition-Latin America (PeC-LA) were included. All centers performed the procedure following the same previously described standardized technique. Inclusion criteria included penile cancer patients with no palpable lymph nodes and intermediate/high-risk disease and those with non-fixed palpable lymph nodes less than 4 cm in diameter. Categorical variables are shown as percentages and frequencies whereas continuous variables as mean and range.

**Results:**

From 2006 to 2020, 210 VEIL procedures were performed in 105 patients. Mean age was 58 (45-68) years old. Mean operative time was 90 minutes (60-120). Mean lymph node yield was 10 nodes (6-16). Complication rate was 15.7%, including severe complications in 1.9% of procedures. Lymphatic and skin complications were noted in 8.6 and 4.8% of patients, respectively. Histopathological analysis revealed lymph node involvement in 26.7% of patients with non-palpable nodes. Inguinal recurrence was observed in 2.8% of patients. 10y- overall survival was 74.2% and 10-y cancer specific survival was 84.8%. CSS for pN0, pN1, pN2 and pN3 were 100%, 82.4%, 72.7% and 9.1%, respectively.

**Conclusion:**

VEIL seems to offer appropriate long term oncological control with minimal morbidity. In the absence of non-invasive stratification measures such as dynamic sentinel node biopsy, VEIL emerged as the alternative for the management of non-bulky lymph nodes in penile cancer.

## INTRODUCTION

Penile cancer (PC) is a rare disease in developed countries with a reported incidence of 1-2 cases per 100,000 males (1). Squamous cell carcinoma, which has several subgroups with various clinical outcomes, accounts for 95% of instances of penile cancer. Penile cancer frequently has a relationship with chronic preputial inflammation brought on by phimosis or lichen sclerosus. Penile cancer risk is decreased by circumcision (hazard ratio: 0.33) (2). HPV was found to be involved in about 40% of penile malignancies (PCs), with HPV 16 being the most common genotype (3). Higher incidences are encountered in some areas of Latin America, Africa and Asia, where it can correspond to 1-2% of malignant diseases in men (4, 5).

Lymphatic spread to inguinal lymph nodes remains as the most important prognostic factor in patients with PC (6). Patients with low volume disease undergoing radical inguinal lymph node dissection have an excellent cancer control and prolonged survival compared to surveillance followed by salvage surgery in case of clinical progression (7).

Despite being recommended by most clinical guidelines, early lymph node dissection in cases of intermediate/high-risk penile cancer is not frequently performed, probably due to high morbidity of the standard procedure (8). Open and VEIL approach have similar safety, overall survival and post-operative outcomes (9).

Over the last 40 years, modified templates, and dynamic sentinel node biopsy (DSNB) were proposed to decrease the morbidity associated with the standard lymph node dissection (10). DSNB false negative rates in large reference centers hangs around 5% (11). Nonetheless, other studies have shown false negative rates as high as 15% (12). In Latin America, DSNB is not commonly utilized, hence, false negative rates of 42% have been described (13).

Endoscopic approach for inguinal lymph node dissection was first described in cadaveric models in 2003 by Bishoff et al. (14). In 2006 To-bias-Machado et al. followed by Sotelo et al. published their first successful experience in patients (15, 16). In 2007, a pilot randomized trial demonstrated the oncological equivalence of VEIL when compared to the open counterpart (17).

After that, several other series and three systematic reviews with pure laparoscopic or robotic techniques have reported further evidence supporting the findings from that landmark comparative study (18–24).

In the present series our goal is to report the larger and longest surgical and oncological outcomes from Latin American patients with pe-nile cancer undergoing VEIL.

## MATERIALS AND METHODS

This is a retrospective and descriptive study of patients operated from 2006 to 2020 (CAAE:46451021.2.1001.5437).

All patients underwent partial or total penectomy and abdominal and pelvic computed tomography scan or magnetic resonance imaging before management of the lymph nodes. Results were reported following the American Joint Committee on Cancer TNM Classification (8th edition) (25). The study was approved by the central Ethics Committees

The indications for VEIL were clinically palpable nodes < 4 cm non-fixed or non-palpable nodes in patients with intermediate or high-risk penile cancer [12]. All patients underwent bilateral VEIL procedure. Patients with an American Society of Anesthesiologist (ASA) Score > 3 were excluded. No patients in this study received neoadjuvant treatments.

We selected 17 centers that routinely utilize a previously reported VEIL standardized technique (15). After training, each institution collects data according to a standardized questionnaire. A minimum of 5 (five) cases per institution was considered to enter in this study. Patients were followed every 3 months in the first 2 years and twice a year for the following 3 years. Physical examination, laboratory testing, and imaging methods were performed according to the EAU guidelines (12).

### Surgical Procedure

### Preoperative workup

Palpable nodes are marked with a skin ink. When nodes are difficult to find, such as in obese patients, the node is marked guided by ultrasound. Intravenous second-generation cephalosporin is administered one hour before the procedure.

### Surgical procedure

Patient is placed in supine position with thigh abducted. The video system must be placed on the opposite side of the limb that is under intervention at the level of the patient's waist. A 3-trocar configuration is applied distal to the femoral triangle. The working space is insufflated with CO2 at 15 mmHg with quick space distention and kept as low as 5-10 mmHg for the duration of the procedure. The main landmarks are the adductor longus muscle medially, the sartorius muscle laterally and the inguinal ligament superiorly. The saphenous vein is located medially and the spermatic cord and the superficial inguinal ring superior-medially. The saphenous vein is dissected and preserved cranially up to the fossa ovalis close to the safeno-femoral junction. Modified template is recommended in order to reduce lymphatic complications. All cases underwent modified ILND technique. Following the fascia lata we identify the femoral vessels that constitute the deep limit for the dissection. All areolar tissue located me-dial to femoral artery must be removed. Small vessels are sealed with harmonic scalpel and control of larger lymphatics is obtained using clips. The specimen is totally dissected after ligation of the proximal portion of the lymphatic tissue at the deep portion of the femoral canal.

### Perioperative care

Prophylactic intravenous antibiotics are administered routinely during hospital stay. In the 15 days postoperative period, patients can undergo early walking and anti-embolic socks. Suction drain is removed when output is less than 50 mL/day. Hospital discharge does not depend on the drain output. It can be removed in the first 7-10 days post operative presentation. Postoperative use of low molecular weight heparin is not standard, as it is indicated according to each institution's protocol. Post operative antibiotics aren't needed.

### Analyzed parameters

Perioperative data such as operative time, 90 postoperative days complications according to Clavien-Dindo classification, hospital stay, lymph node yield, days to remove drain, number of positive nodes, local and systemic recurrence and trocar recurrences were reported. Cancer specific (CSS) and overall survival (OS) were calculated for the pathological nodal stage (pN). Survival outcomes were compared with contemporary series of open surgery to estimate oncological control with VEIL.

Lymphedema was assessed by the same assistant physician according with physical exam and tonometer. Physiotherapy and compression rates also were evaluated.

## Statistical Analysis

CSS was calculated using the Kaplan-Meier method, which was stratified by lymph node status (positive vs negative) and lymph node staging (pN1, pN2, or pN3). Survival time was calculated from the time of surgery to death or censored at the date of most recent follow-up for patients who did not die. Univariate Cox regression analysis was used to determine differences in cancer-specific death risk according to lymph node status and lymph node staging. All statistical analyses were performed using SPSS version 24 (IBM SPSS Statistics Subscription for Mac OS).

## RESULTS

All 17 centers sent 127 cases to our database, however only 105 with complete follow-up were included in this report. Procedures were bilateral in 105 patients, with a total of 210 groins undergoing VEIL. Median follow-up was 10 years (1-14 years). Clinical characteristics of our sample are shown in Table-1. Mean age of the patients included was 58 (45-68) years old.

**Table 1:** Patient characteristics (n = 105 patients).

		n
Age Mean (Range)	-	58 (45-68)
**BMI**	< 25	19
	25-30	63
	> 30	23
**ECOG**	0	85
	1	20
Tumor Pathological Classification (pT)	T1	4
Age Mean (Range)	-	58 (45-68)
**BMI (%)**	< 25	19 (18.1)
	25-30	63 (60)
	> 30	23 (21.3)
**ECOG (%)**	0	85 (81)
	1	20 (19)
**Tumor Pathological Classification (pT) (%)**	T1	4 (3.8)
	T2	23 (21.9)
	T3	78 (78)
**Lymph Node Clinical Classification (cN)I(%)**	N0	71 (67.6)
	N1	28 (26.7)
	N2	6 (5.7)
**EAU Risk Group for N0I (%)**	Intermediate	8 (11.3)
	High	63 (88.7)
**Cubillas Risk ScoreI (%)**	Intermediate	10 (14.1)
	High	61 (85.9)
CT Scanl (%)		90 (85.7)
MRI (%)		15 (14.3)

BMI = Body mass index; ECOG = Eastern Cooperative Oncology Group; EAU = European Association of Urology; CT = Computed tomography; MRI = Magnetic resonance imaging

Perioperative parameters are described in Table-2. VEIL was performed in 210 groins (105 patients). Mean operative time was 90 minutes (60-120). No conversions were reported. Mean hospital stay was two (1–3) days. Four (3.8%) hospital readmissions were necessary, one due to skin necrosis, two infected lymphoceles. Mean number of retrieved lymph nodes (range) was 10 (6–15).

**Table 2:** Perioperative data and post operative complications according with Clavien-Dindo Classification of 210 VEIL procedures.

	Mean	Range			
Surgical time (minutes)	90	60-120			
Conversion	0	0			
Number of Lymph nodes retrieve	10	6-15			
Drainage time (days)	7	3-21			
Hospital Stay (days)	2	1-3			
Hospital Readmission (%)	4 (3,8)				
System	n	Overall (%)	Clavien 1 or 2 (n)	Clavien 3 or 4 (n)	Clavien 3 or 4 (%)
Skin	10	4.8%	9	1	0.5%
Lymphatic	18	8.6%	16	2	1.0%
Vascular	0	0	0	0	0
Overall	28	13.3%	25	4	1.9%

Postoperative complications according to the Clavien-Dindo system are reported in Table-3. Skin complications corresponded to 4,8 % of the complications with only one (0.5%) severe case (Clavien-Dindo grade 3 or 4).

**Table 3:** VEIL 10-year Overall and Cancer Specific (CS) Survival Rate according with Regional Lymph Node Disease (n = 105 patients).

Histopathological Stage (pN)	N	Overall Deaths (n)	Overall Survival (%)	CS* Deaths (n)	CS* Survival (%)
0	66	7	89.4%	0	100.0%
1	17	4	76.5%	3	82.4%
2	11	6	45.5%	3	72.7%
3	11	11	0.0%	10	9.1%
**Total**	**105**	**28**	**73.3%**	**16**	**84.8%**

Lymphatic complications corresponded to 8.6% (14 lymphocele and 4 lymphedema). Three cases did not solve with manual compression, weight loss and exercises and needed further physiotherapy. Major complications in two (1.0%) severe cases needed surgical lymphocele drainage.

Sixteen patients (15.2%) died from penile cancer disease. Twelve (11.4%) died from other non-penile cancer causes. The median follow-up for cancer-free patients was 10 years. Four (3.8%) presented with pT1 disease, the majority (96.2%) presented with pT2 or pT3 disease. Seventy-one (67.6%) presented with no palpable lymph nodes, whereas 63 (88.7%) were high risk and eight (11.3%) had intermediate risk to develop regional metastasis, according with the EUA risk stratification. Similar results were observed in the Cubillas Risk Score.

**Figure 1:**
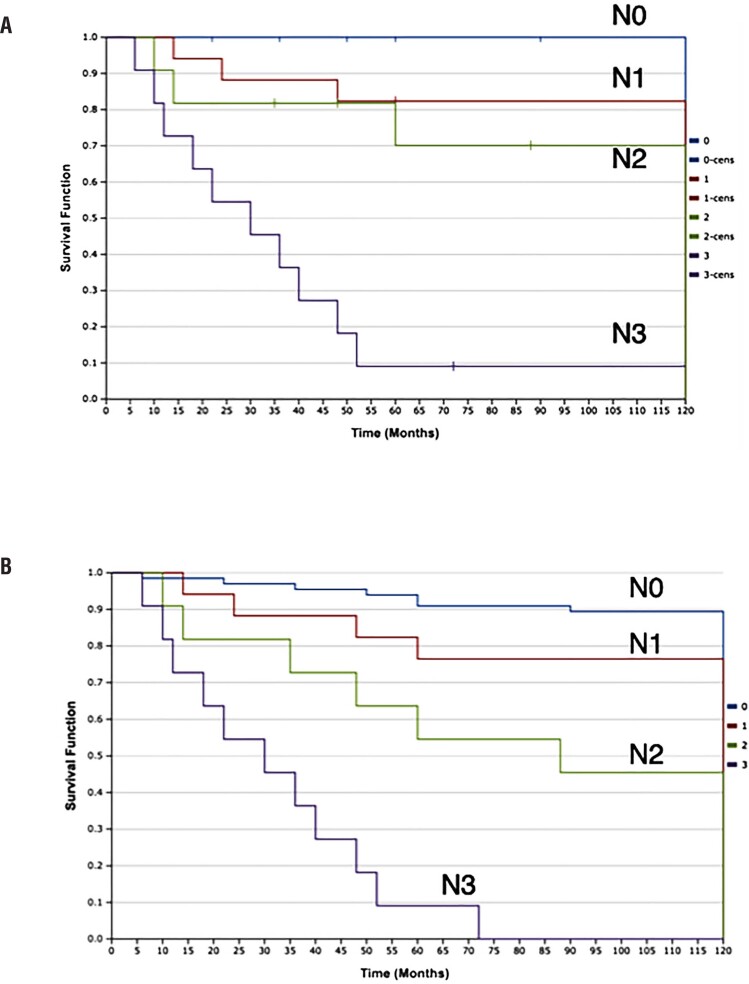
Kaplan-Meier curves of cancer specific survival and overall survival.

Positive lymph nodes were observed in 26.7% of cN0 patients and negative lymph nodes were noticed in 41.1% in cN+ group. Cancer specific survival (CSS) and Overall Survival (OS) were reported in the Figure-1. In this series inguinal recurrence was 3,8% and disease progression were noticed in 41% of the pN+ group (16/39), with 3 cases in pN1 group, 3 cases in pN2 group and 10 in pN3 group. Ten year cancer specific survival was 84,8 % and 10-year overall survival was 73,3% (Figure-1). In this series inguinal recurrence was 3.8% and disease progression was noticed in 41% of the pN+ group (16/39), with 3 cases in pN1 group, 3 cases in pN2 group and 10 in pN3 group.

Ten-year cancer specific survival was 84.8 % and 10-year overall survival was 73.3% (Figure-1).

Survival and histopathological analysis after bilateral VEIL are reported in Table-3. No portal seeding was documented. One-hundred-five patients with cN0-N2 underwent bilateral VEIL, whereas after histopathological analysis, sixty-six revealed to be pN0, seventeen pN1, eleven pN2 and eleven pN3. The 10-year OS and CSS regarding regional lymph node disease are reported at Table-3. CSS for pN0, pN1, pN2 and pN3 were 100%, 82.4%, 72.7% and 9.1%, respectively.

## DISCUSSION

Inguinal management may vary according with the presence or absence of palpable lymph nodes. Those without palpable nodes and high risk of inguinal involvement need any kind of inguinal screening. In countries with high volume reference centers DNSL can achieve excellent oncological outcomes with false-negative rates of 5% regarding the low complication rates (10). In positive cases, ILND is mandatory. For patients with palpable nodes, EAU guideline recommends image staging with PET-CT and ILND as treatment (11). In Latin America there is no report with good outcomes with DNSL (12) and PET-CT is not wide available to PC patients. In this scenario, the ILND is the standard of care in patients with risk of nodal spread.

After the period of the initial learning curve, VEIL has been gaining growing acceptance. One of the strengths of our study is that the pioneers of VEIL technique served as mentors for all surgeons performing the procedure in this study, ascertaining standardization of the technique across all participating centers. Our perioperative parameters showed acceptable operative time and no conversions at the 17 centers. The patients were discharged after a median of 2 (range 1- 5) days of hospital stay. A recent metanalysis including 290 patients in 10 comparative studies reports reduced morbidity of VEIL when compared to the open procedure. Oncological outcomes were similar in a short-term follow-up (25). Other two systematic reviews confirmed these results (26, 27).

Small number of subjects (since this is a rare disease) and absence of long term follow up is a major limitation of previous VEIL studies. So far, the studies with the longest follow-up reported good oncological outcomes after a median of 16 to 55 months (23, 24). In the present study we report the outcomes after a median of 120 months, which is the longest follow-up published so far.

Reduced skin morbidity is the most robust advantage observed in VEIL (4.8%) compared to open surgery series (30-60%) (5–8) followed by an important decrease in lymphatic morbidity. Some preliminary studies of VEIL reported higher incidence of lymphocele probably due to use of energy to seal lymphatic vessels. We refined our technique with caution to identify lymphatic vessels and clip the distal extremity instead of cauterizing it. This modification might have the potential to reduce the lymphatic events. Future comparative studies may prove this concept. Lymphedema and lymphocele are initially managed with conservative treatment with a low-fat diet, low hydric oral intake associated with compression and physiotherapy. A recent metanalysis shown reduced lymphoedema with VEIL vs O-ILND (OR=3.23, 95% CI [1.51, 6.88], P=0.002), however with no difference in lymphocele (OR=0.83, 95% CI [0.31, 2.23], P=0.720) (25). Surgical drainage along with third or fourth generation cephalosporin is administered when an infected lymphocele is suspected. Reoperation with surgical drainage and lymphatic control was carried out in only 2 cases.

Oncological principles include removal of all tissue superficial to the fascia lata inside the limits of the femoral triangle and medial to the femoral artery under fascia lata including the oval fossa and the femoral channel. To achieve complete removal of lymphatic nodes, ultrasound imaging and palpation before and after procedure were important to avoid leaving some “lost” nodes at the superficial area. Dissection of deep femoral triangle was easier and vascular accidents were extremely rare and controlled laparoscopically, with no conversion needed. Mean number of retrieved nodes in this report was 10, which is comparable to the number of retrieved nodes reported in the open approach series. Most series showed that endoscopic techniques can remove an equivalent number of nodes when compared to the standard open surgery (4–8).

The number of metastatic lymph nodes reflects severity of the disease and influences survival. Some authors tried to discriminate between good and poor risk groups of patients. A significant difference was observed between 1 to 3 positive inguinal nodes vs. 4 or more nodes, in accordance with Li et al., in a 2018 retrospective series of 196 patients that demonstrated similar outcomes (28). In this current study we have found similar results. Most patients with pN3 disease had the worst CSS with early recurrence and poor survival two years after the surgery, with a CSS of only 10% after 5 years of follow up. Patients with pN1 and pN2 disease had similar OS and CSS rates.

Previous reports of open surgery showed positive lymph nodes in 20-30% of high-risk patients with no palpable nodes (cN0) (5–8). In the present series we found 26.7% of cN0 patients with positive inguinal nodes after histopathological analysis. In those with palpable nodes, a more intensive preoperative workup may be the key point to identify the high-risk patient in order to offer neoadjuvant (NAC) protocols to avoid upstaging ILDN/VEIL (29). In this series all NAC cases were excluded due lack of standardized protocols; some cases received taxane and others not, and different time to treat, many received NAC before and others after the penectomy. A future collaborative protocol from the Penile Cancer Collaborative Coalition-Latin America (PeC-LA) may answer this question.

Overall inguinal recurrence after VEIL was rare (3.8%). Pelvic and systemic recurrence are unfortunately higher in patients with extracapsular extension (pN3). Even when considering salvage chemo or radiotherapy mean survival in this situation was poor (6-9 months). No cases of trocar seeding were documented. We hypothesize that the outcomes were more correlated with aggressive biology of disease than with surgical technique.

The majority of patients of our series presented with low volume disease. Initial inguinal disease and adjuvant chemotherapy for positive inguinal nodes can explain the excellent survival curves observed in the present study. No patients with neoadjuvant treatment were considered in this study. Adjuvant chemotherapy was carried out for all pN2+ patients. Adjuvant radiotherapy was considered in the palliative care scenario and/or pN3 patients with local or systemic recurrence.

The findings of our study must be analyzed in light of some limitations. First, there are limitations due to the retrospective design of this study. Second, due to the rarity of PC, the study encompassed a long period of time with possible heterogeneity in the management of patients across different periods. Third, due to the learning curve of a new procedure, the initial cases at each participating center might have had a negative impact on the outcomes of our study. Fourth, despite standard follow-up protocol, retrospective studies may have underreported complications. We expect that with further dissemination of the technique to other centers that treat PC and with further improvements in the surgical technique we will see even better outcomes in the near future.

In the last few years, we have observed an increase in the adoption of a Robotic Assisted- VEIL (R-VEIL) approach, probably stimulated by a seemingly shorter learning curve when compared to pure laparoscopic VEIL (19, 27). Currently R-VEIL and conventional VEIL outcomes seem to be quite comparable. Further improvements associated with robotic platforms are expected for the near future. We speculate that R-VEIL will be standard of care in the next decade when inguinal lymph node dissection will be expanded for cases with N0-N2 disease. Future randomized studies will be important to demonstrate clinically important advantages of R-VEIL over pure VEIL.

## CONCLUSION

In patients with low volume disease VEIL seems to offer excellent long term oncological control with reduced morbidity, specially the skin complications. In the absence of non-invasive stratification such as dynamic sentinel node biopsy, VEIL emerges as an alternative staging tool with simultaneous nodal treatment to this aggressive disease.
